# RLIMS-P: an online text-mining tool for literature-based extraction of protein phosphorylation information

**DOI:** 10.1093/database/bau081

**Published:** 2014-08-13

**Authors:** Manabu Torii, Gang Li, Zhiwen Li, Rose Oughtred, Francesca Diella, Irem Çelen, Cecilia N. Arighi, Hongzhan Huang, K. Vijay-Shanker, Cathy H. Wu

**Affiliations:** ^1^Center for Bioinformatics and Computational Biology, University of Delaware, Newark, DE 19711, USA, ^2^Department of Computer and Information Sciences, University of Delaware, Newark, DE 19711, USA, ^3^Lewis-Sigler Institute for Integrative Genomics, Princeton University, Princeton, NJ 08544, USA, ^4^Structural and Computational Biology Unit, EMBL (European Molecular Biology Laboratory), 69117 Heidelberg, Germany, ^5^Department of Biochemistry, Molecular and Cellular Biology, Protein Information Resource, Georgetown University Medical Center, Washington, DC 20007, USA

## Abstract

Protein phosphorylation is central to the regulation of most aspects of cell function. Given its importance, it has been the subject of active research as well as the focus of curation in several biological databases. We have developed **R**ule-based **L**iterature **M**ining **S**ystem for protein **P**hosphorylation (RLIMS-P), an online text-mining tool to help curators identify biomedical research articles relevant to protein phosphorylation. The tool presents information on protein kinases, substrates and phosphorylation sites automatically extracted from the biomedical literature. The utility of the RLIMS-P Web site has been evaluated by curators from Phospho.ELM, PhosphoGRID/BioGrid and Protein Ontology as part of the BioCreative IV user interactive task (IAT). The system achieved F-scores of 0.76, 0.88 and 0.92 for the extraction of kinase, substrate and phosphorylation sites, respectively, and a precision of 0.88 in the retrieval of relevant phosphorylation literature. The system also received highly favorable feedback from the curators in a user survey. Based on the curators’ suggestions, the Web site has been enhanced to improve its usability. In the RLIMS-P Web site, phosphorylation information can be retrieved by PubMed IDs or keywords, with an option for selecting targeted species. The result page displays a sortable table with phosphorylation information. The text evidence page displays the abstract with color-coded entity mentions and includes links to UniProtKB entries via normalization, i.e. the linking of entity mentions to database identifiers, facilitated by the GenNorm tool and by the links to the bibliography in UniProt. Log in and editing capabilities are offered to any user interested in contributing to the validation of RLIMS-P results. Retrieved phosphorylation information can also be downloaded in CSV format and the text evidence in the BioC format. RLIMS-P is freely available.

**Database URL:** http://www.proteininformationresource.org/rlimsp/

## Introduction

The reversible phosphorylation of proteins is central to the regulation of most aspects of cell function. The flow of molecular information through signaling pathways frequently depends on protein phosphorylation mediated by specific kinases that recognize and phosphorylate-specific sites in the target proteins (1). In many cases, deregulation of the kinase-substrate network has been linked to diseases, including cancer (2). Thus, protein phosphorylation has been an active research area, and relevant findings through studies have been curated in several databases, such as Phospho.ELM (3), PhosphoGRID (4, 5), PhosphoSitePlus (6), Protein Ontology (PRO) (7) and the UniProt Knowledgebase (UniProtKB) (8).

Scientific publications are the primary source for gathering research findings, e.g. when approaching a new problem or developing and/or supporting working hypotheses. An expert review of research literature is also a key step in the biocuration workflow in well-curated high-quality databases such as those mentioned above. To support the efficient identification and review of phosphorylation-related literature, we have developed a rule-based information extraction (IE) system, named RLIMS-P, a **R**ule-based **L**iterature **M**ining **S**ystem for protein **P**hosphorylation (9, 10). RLIMS-P is an online text-mining tool that provides an interface to identify articles relevant to protein phosphorylation, and presents information on protein kinases, substrates and phosphorylation sites extracted from the biomedical literature. RLIMS-P has been used to support curation of phosphorylation information and the construction of protein phosphorylation networks (3, 11–13). It also has been integrated as a system module to provide phosphorylation information necessary for another text-mining system, eFIP, which extracts the functional impact of phosphorylation events (14, 15).

The RLIMS-P online tool relies on three components: (i) RLIMS-P version 2.0, which offers enhanced scalability, generalizability and maintainability when compared with the original system (version 1.0). Extraction performance of RLIMS-P 2.0 was previously evaluated on several different corpora, and the F-scores were in the 90% range (16). RLIMS-P 2.0 has been applied to full-scale mining of all MEDLINE abstracts (16); (ii) the database, which stores the phosphorylation information extracted from full-scale literature mining, along with gene normalization results (linking of substrate/kinase mentions to UniProtKB identifiers); and (iii) the web interface, which enables the users to find articles relevant to phosphorylation and associated information via keyword searches similar to a PubMed search, as well as by selected species, or a list of PubMed IDs (PMIDs). The results (PMID, kinase, substrate, sites and text evidence) are displayed in sortable tables, which are downloadable in CSV format for further research. Editing functionality is also available for registered users. The RLIMS-P system is hosted on the iProLink Web site (17), a public resource to facilitate text mining at the Protein Information Resource (PIR) (18).

The utility of RLIMS-P for biocuration was evaluated in the BioCreative IV user interactive task (IAT). Expert curators from Phospho.ELM, PhosphoGRID/BioGRID and PRO used the Web site to review literature and curate the phosphorylation information. The curators provided real-time annotation results that were used to evaluate the system performance of RLIMS-P, along with feedback on the system via a user survey, which was used to improve the Web site usability. In this article, we report on the evaluation task conducted in the BioCreative IAT and then describe the RLIMS-P interface, highlighting the modifications introduced based on the user feedback from this activity.

## Material and methods

### RLIMS-P

#### RLIMS-P version 2.0

The RLIMS-P 2.0 system consists of several customized modules for biomedical text processing, including (i) a shallow parser based on part-of-speech tags and hand-crafted rules for syntactically analyzing input text, e.g. detecting noun phrases and verb group phrases, (ii) a term classifier that annotates noun phrases with predefined semantic categories, such as protein, protein part and chemical, also using rules defined over the headwords of the phrases, their affixes and the words surrounding these phrases, (iii) a pattern-based IE engine that extracts phrases referring to target entities (kinase, substrate and site of phosphorylation) and (iv) an additional IE component that identifies a phosphorylation event reported across multiple clauses and sentences [see (10, 16) for details].

A major enhancement in RLIMS-P 2.0 is the design of the IE engine that eases the management (i.e. adding and editing) of the IE patterns. The new system implements simpler IE patterns, while allowing those patterns to be combined to cover a large number of diverse expressions used to report protein phosphorylation in the research literature. Additionally, sentence simplification techniques particularly effective for biomedical IE (19) were exploited to improve the coverage of the implemented IE patterns. These changes have simplified the system design and contributed to the system’s generalizability and maintainability. F-scores obtained by RLIMS-P 2.0 ranged from 0.83 to 0.98 when evaluated on benchmark corpora, such as the BioNLP GENIA event extraction corpora (20), and an in-house annotated corpus (16).

#### Database

RLIMS-P 2.0 is applied to full-scale text mining of MEDLINE abstracts (16). We first filter articles potentially containing phosphorylation information based on the presence of the RLIMS-P trigger words in the title and abstract, where those trigger words include phosphorylation, phosphorylate(s), phosphorylated, phosphoprotein(s), phospho amino acid(s), among others (see list provided in Supplementary Material).

To support quick online retrieval, the extracted phosphorylation information (kinase, substrate, site), along with text evidence, has been stored in a back-end database (as part of the iPTMnet enzyme-substrate database) (12). Normalization of protein names obtained by GenNorm (21) is integrated in this database. Additionally, we check the PMIDs in the database against those cited in UniProtKB bibliography (8) to provide links to curated data and assist in the normalization process. The database, initially built using the 2013 release of the MEDLINE archive, contains phosphorylation information extracted from 165 840 abstracts, and links to 43 329 UniProtKB entries. The database is updated weekly in sync with PubMed.

#### Web interface

An enhanced Web interface has been designed to access the integrated phosphorylation information based on RLIMS-P 2.0 and other resources, such as normalization and bibliography mapping. The help document of the Web site is available online. The Web service is provided through an Apache HTTP server, and the Web programs are written primarily in PHP and JavaScript. The Web site supports major Web browsers (including Google Chrome, Mozilla Firefox, Internet Explorer and Safari). Phosphorylation information can be downloaded in CSV format, or evidence text in the ‘BioC’ standard corpus annotation format (22). The Web interface is publicly available for searching, browsing and downloading of information, and the functionality for online curation, including the additional editing functionality aimed at validating text-mining results, is also available for logged-in users. The Web site provides options to restrict the search results to selected species and also to filter out review papers. Both functionalities are provided by leveraging PubMed query. By default, the Web site will forward the user query to PubMed to obtain related PMIDs. If one or more species are selected, the corresponding species names and synonyms are added to construct the PubMed query. For example, if the user selects species ‘Human’, the query sent to PubMed would be ‘user_query[TIAB] AND (Homo sapiens [TIAB] OR human [TIAB] OR Modern Man [TIAB] OR Humans [MeSH Terms])’. We use the PubMed search field ‘Publication Type [PT]’ to filter out review papers, i.e. ‘user_query[TIAB] AND NOT Review [PT]’. In addition, RLIMS-P Web site now includes results of full-text articles if the articles are available in the PubMed Central (PMC) open-access subset. As for the full-text articles, the sections most relevant to experimental results (Abstract, Results, Figure legend and Discussion) are presented.

### Evaluation tasks

As part of the BioCreative IAT, RLIMS-P was evaluated by three expert curators from Phospho.ELM, PhosphoGRID/BioGRID and PRO. All the curators were experienced in annotating kinase-substrate relations. In conducting this evaluation study, curation guidelines were developed. The guidelines detailed the types of entities to be annotated (kinase, substrate and site) and how the detected proteins should be normalized to UniProtKB entries. The guidelines included examples and exercises for the curators to be familiar with the curation criteria and the Web interface. Importantly, the RLIMS-P task was aligned with the real curation task, that is, the curators were asked to validate information at the document level, not at the sentence level, and consider only experimentally determined data. The annotation guidelines can be accessed by curators after logging in or, alternatively, via the following link http://research.bioinformatics.udel.edu/rlimsp/files/RLIMSP_guidelines.pdf. Here we summarize some important aspects of the annotation task.

#### What is annotated as kinase, substrate and site?

A kinase is the catalyst of the phosphorylation reaction, whereas the substrate is the entity that is phosphorylated. Kinases or substrates could be individual proteins (e.g. Crm1), protein complexes (e.g. CDK1-cyclin-B) or a group of related proteins (e.g. Src kinases). All these entities should be annotated, and proteins need to be linked to UniProtKB identifiers. The abstract of PMID:23729730 provides an example where a substrate is a protein (Crm1) and the kinase is a complex (CDK1-cyclin-B): ‘Here, we report that human Crm1 is phosphorylated at serine 391 in mitosis by CDK1-cyclin-B’. A site in RLIMS-P can be a residue type (Serine, Threonine, Tyrosine), a specific residue (Ser-391) or a protein region or domain (C-terminal domain). The curator should annotate the most specific site possible. Therefore, the annotation for the example above should be Substrate: Crm1, Kinase: CDK1-cyclin-B, Site: Ser-391.

As previously mentioned, we asked curators to provide information about substrates, kinases and sites at the document level. If there are multiple sentences conveying the same information, the selection of only one is sufficient. In RLIMS-P, the output is presented in this way; identical outputs are collapsed so the curator does not have to spend time in corroborating all sentences. Occasionally, textual variations of an entity may artificially create redundant information. For example, in PMID:22126602, sentences such as ‘In the experiments using COS-7 cells expressing FLT3/ITD and Wt or mutant beta-catenin, FLT3/ITD phosphorylated Y654, and this residue was essential for beta-catenin's nuclear localization by FLT3/ITD.’ and ‘In vitro kinase assays, using recombinant FLT3 and biotinylated beta-catenin peptide including Y654 showed that FLT3 directly phosphorylated Y654 of beta-catenin’. Both indicate that Y654 in beta catenin is phosphorylated by FLT3. However, RLIMS-P presents two lines of annotation with FLT3/ITD as kinase in one case and FLT3 in the second one. Therefore, curators need to select only one.

Curators were also requested to validate only phosphorylation information with experimental support. This would be the case for PMID:23382206 in sentence ‘Here we show, in the developing chick neural tube, that phosphorylation of Sox9 on S64 and S181 facilitates its SUMOylation, and the phosphorylated forms of Sox9 are essential for trunk neural crest delamination.’, and not the case for PMID:21443865 where phosphorylation is mentioned in the pass ‘Glycogen synthase kinase-3beta (GSK-3B) phosphorylates tau protein, and increased GSK-3B expression has been associated with neurofibrillary tangles’.

For individual protein mentions, curators were asked to link kinase and substrate mentions to UniprotKB accessions, whenever possible. RLIMS-P suggests in many cases some UniProtKB entries that potentially correspond to the protein mentions in the abstract. In many cases, consultation with full text is needed, and RLISM-P provides a link to full text if the article is open access.

#### Information extraction

Each curator was provided with a set of 50 PMIDs (two sets of 25 PMIDs), specifically tailored to their curation tasks:

##### Data set 1

The first data set, prepared for the Phospho.ELM curator, was compiled for any kinase-substrates relation reported in articles published in 2013. The selection of 50 PMIDs were based on the PubMed query, (“2013/01/01”[Date - Publication]:“3000”[Date - Publication]) AND kinase AND phosphory*, and then inspected to confirm that the contents were appropriate for the curation task.

##### Data set 2

The second data set, prepared for the PhosphoGRID/BioGRID, included articles with phosphorylation information on yeast, published between 2012 and 2013. The selection of 50 PMIDs was based on the PubMed query, (“2012/01/01”[Date - Publication]:“3000”[Date - Publication]) AND (saccharomyces OR yeast) AND phosphory*, and then also confirmed for appropriate contents.

##### Data set 3

The third data set, prepared for the PRO curator, contained abstracts collected from literature related to transient potential receptors as well as 11 abstracts from data set 1 and 36 from data set 2.

The curators were requested to identify kinase, substrate and phosphosite for each detected phosphorylation event at the document level and to normalize the protein information when possible. The curators performed this task using RLIMS-P on half of the provided collection (i.e. a set of 25 MEDLINE records) and did the same on the other half without using the Web site. Besides reporting the curation time for each setting, they were also requested to answer questionnaire created by the BioCreative organizers concerning the utility of the tool and the user experience. When the curation was conducted on the RLIMS-P Web site, the annotation results could be recorded online. These results were used to evaluate the automated extraction performance of RLIMS-P. The performance was measured by precision, recall and F-score as follows:

Precision=TP/(TP+FP),

Recall=TP/(TP+FN),

F-score=2·Precision·Recall/(Precision+Recall),

where TP, FP and FN are numbers of true-positive results (correctly extracted phrases), false-positive results (phrases that should not have been extracted) and false-negative results (phrases that should have been extracted), respectively. When the curation was conducted without using the Web site, a spread sheet was provided to record the curation results. These results were used to calculate the inter-annotator agreement rate measured by F-score as described below.

#### Inter-annotator agreement

A set of 34 abstracts annotated during the evaluation task was reviewed by two curators, and most of those were annotated without using the RLIMS-P Web site. These abstracts were gathered to calculate the inter-annotator agreement rate using an F-score. Here, annotations by one curator were regarded as gold-standard, and the ‘performance’ of the other curator measured using F-score. The F-score is the same if either annotator is selected as a gold-standard annotator because the selection of this role only switches the precision and recall, and hence, the resulting F-score remains the same.

#### Document triage tasks

The curation task described above aligns with the design of the IE mechanism implemented in RLIMS-P. However, the practical utility of RLIMS-P is not limited to the extraction of entities, but also includes its use in searching and retrieving relevant literature containing the phosphorylation information. In fact, selecting the set of relevant papers to curate is an important step in the curation workflow (23). To confirm the utility of the Web site for document retrieval, we additionally requested two curators to submit five PubMed keyword queries on the Web site and to manually confirm the accuracy of the retrieved results.

## Results and discussion

In this section, we will first present the results on the evaluation of RLIMS-P in the BioCreative IAT, then we will describe the interface that was improved on user feedback during this activity, with practical application for general research beyond biocuration.

### RLIMS-P evaluation in BioCreative IAT

RLIMS-P was evaluated for the extraction of entities (IE) and also for retrieval and filtering of relevant documents (document triage).

#### Information extraction

Each of the three curators has reviewed a set of 50 MEDLINE records. A half of the records were reviewed on the RLIMS-P Web site (three sets of 25 MEDLINE records) and used to evaluate the performance of RLIMS-P. These sets were suitable for the performance evaluation because the correctness of the automated extraction can be directly judged by the edit status of the annotated entities, i.e. an entity extracted by RLIMS-P was either validated (true positive) or rejected (false positive) by the curator, and an entity missed by the system was manually added during the curation task (false negative) (We are aware of the potential bias in using such pre-annotated text for the evaluation study, in particular potentially inflated recall performance due to the curators’ oversight of annotation instances. We, however, believe that the fraction of such instances was negligible because the expert curators would seldom miss the sought information in the abstracts. Readers, however, may refer to our evaluation studies reported in our prior publication, in which diverse corpora annotated independent of RLIMS-P were used.). The evaluation results are shown in Table 1.

**Table 1.** Evaluation of RLIMS-P on manual curation tasks conducted by three curators

Kinase			Substrate			Site		
Precision	Recall	F-score	Precision	Recall	F-score	Precision	Recall	F-score
0.69	0.86	0.76	0.82	0.95	0.88	0.89	0.95	0.92

While the annotated data set was small compared with those used in the earlier evaluation studies of RLIMS-P 2.0 (16), the F-scores for substrate and site were comparable (∼0.9). As for kinase, closer inspection of the results revealed two issues resulting in lower scores. One of them was exclusively related to curation aspects. RLIMS-P was designed for a general user to extract phosphorylation relations stated in the text, while the current evaluation was intended for actual database curation. For curation purposes, RLIMS-P extractions would be marked as false–positive results if the article did not contain any experimental evidence. This was particularly evident for the kinase information. Also, most relations extracted from review articles would be treated as errors, as these rarely contain actual experiments. The second issue was that, during BioCreative IV, RLIMS-P was configured to report more probable kinases, i.e. to weigh the recall more than the precision, because kinases are reported less frequently. This also contributed to lower precision for kinase. The results suggest that to tailor RLIMS-P for database curation, we should provide an option to filter out review articles, as well as a functionality to recognize underlying experimental evidence for extracted relations. We have now introduced a checkbox to exclude review articles in the search to solve the former issue.

All curators participating in the BioCreative IAT were requested to record the time they spent on the curation task with and without using the text-mining system. In our experiments, two curators reported no difference in curation time using the text mining system, while one curator reported a reduction of 2 h (from 5 to 3 h) in curation time. This apparent negative result from two curators is not consistent with the positive survey feedback that the system received (Table 2). The survey included a set of questions for six topics on a 5-point rating scale, with 1 being the most negative and 5 the most positive response. The median for all topics ranged between 4 and 5. The complete survey responses can be found in (Supplementary Table S1).

**Table 2.** Subjective evaluation of RLIMS-P conducted by three curators

Subjective measure	Median	25% quartile	75% quartile
Overall impression	4	4	5
Comparison to other systems	4	4	5
Task completion	4	4	5
Design	5	4	5
Learnability	4	4	5
Usability	4	4	5

To understand these somehow conflicting results, we reviewed the required curation steps with the curators. The curators described spending most of their reported time on assigning UniProtKB identifiers to protein mentions and, hence, on reading full-text articles, rather than the validation of the RLIMS-P extraction. RLIMS-P focuses on specific tasks within the phosphorylation curation workflow, while the entire curation workflow involves several steps, namely, validation of extracted entities by RLIMS-P, finding the source species and linking to the UniProtKB identifier. Our system provided limited support for protein normalization, e.g. GenNorm was applied only to abstracts, not to full text in this experiment. Therefore, the curation steps exclusively related to RLIMS-P phosphorylation extraction are not readily separable from the complete workflow, and there is no simple way to quantify in this experiment the extent to which RLIMS-P module contributes in the real-world curation work. As stated above, however, the system received highly positive feedback in the survey and also positive remarks from the three curators who conducted the realistic curation work and are currently considering adopting RLIMS-P as part of their database curation workflow. We found such qualitative evaluation was useful for the further development of the system, and have already incorporated some of their suggestions in the Web interface (see RLIMS-P interface).

Some abstracts annotated during the evaluation task, mostly among those that were annotated without using the Web site, were reviewed by two curators. They were used to calculate the inter-annotator agreement rate. High agreement rates were achieved as measured by F-scores of 0.83, 0.90 and 0.95 for kinase, substrate and site, respectively. Interestingly, the agreement measurements follow the expected complexity in identifying the different types of entities, i.e. kinases were the most difficult to agree on, especially in judging the kinase’s direct involvement in the phosphorylation event.

#### Document triage tasks

Besides the main annotation task, two of the curators (F.D. and I.Ç.) were additionally requested to pose several PubMed queries in retrieving MEDLINE records pertaining to their curation domain. This evaluation task aimed at assessing the utility of RLIMS-P for document triage. Five queries used for this evaluation task were as follows: ‘Mass Spectrometry mouse, Breast cancer, Structure AND interaction, PAR4, P2Y12’. Ten MEDLINE records retrieved for each query were manually judged to estimate the precision (in this experiment, only precision could be calculated, as the calculation of recall would require a huge set of random abstracts to be manually annotated. Evaluation measures commonly used for document retrieval, such as mean average precision, is not applicable to the current experiment because RLIMS-P does not rank abstracts, but instead it classifies them as relevant or irrelevant) of the retrieval performance and the averaged precision was found to be 0.88 (0.9, 0.9, 0.7, 1.0 and 0.9, respectively, for the five queries). The error was primarily because of the extraction of information from review articles, similar to the errors in the IE task, which should be pre-filtered according to the specified article type in the curation work. Curators highly appreciated RLIMS-P as a tool for triaging documents because it quickly provided a set of relevant articles for curation.

### RLIMS-P interface

The interface used in the user testing has been described in (24). In this section, we describe the enhanced RLIMS-P interface, which provides multiple functionalities for input query and output navigation, and highlight the changes introduced based on feedback from the IAT, including extension to full-length open access articles, the highlighting of species mentions in text, the restriction to species in the search, the option to exclude review articles and ability to annotate mentions of complex subunits and protein families (for logged in users). Using beta catenin as an example, we demonstrate the type of information that can be quickly gathered in RLIMS-P. Beta catenin has roles in both cell adhesion and transcription co-activation. Loss of beta catenin adhesion function and/or abnormal increase in its transcriptional activity have been implicated in cancer (25). Beta catenin’s function is regulated by phosphorylation. We will describe functionalities of the Web site in finding information about all kinases that phosphorylate beta catenin and the sites (when known) in articles related to cancer.

#### Input page

**Figure 1.**
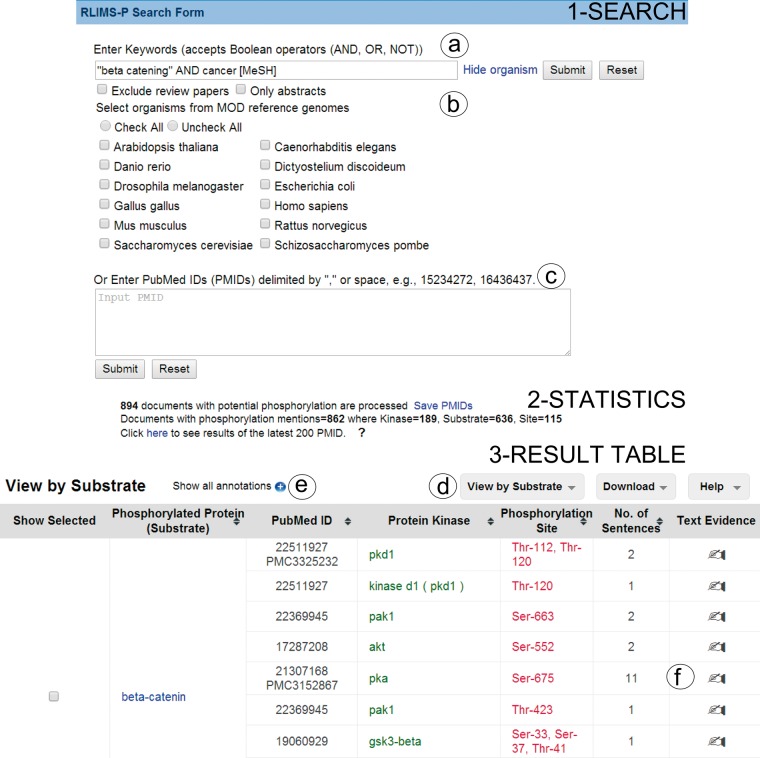
Snapshot of RLIMS-P interface. Search page (1-Search) and result page with statistics (2-Statistics) and table (3-Result Table). The search interface allows queries based on keywords (**a**) with selection of species and/or abstracts only search and possibility to exclude review articles (**b**), or, alternatively, enter a list of PMIDs (**c**). The result table displays the text-mining results along with functionalities (**d**), such as multiple views of the result table (by PMID, by substrate or by kinase), download of table in tab-delimited format, link to help document, expansion of text-mining result by clicking on the plus icon (**e**) and links to text evidence page via the hand icon (**f**). For display convenience, the result table only shows a small subset of the full table.

RLIMS-P online tool offers two forms of input (Figure 1 Search): (i) the input of keywords in PubMed query style (Figure 1a) and (ii) a list of PMIDs (Figure 1c). Based on the user feedback, we introduced options to exclude review articles, as these rarely provide direct experimental evidence, to restrict the query to a particular species (e.g. PhosphoGrid captures information about phosphorylated proteins in yeast) or set of species from a list of model organisms (Figure 1b), and an option to run RLIMS-P in abstracts only. Following our example, we used ‘ “beta catenin” AND cancer [MeSH]’ as a query to find the relevant articles for beta catenin phosphorylation in cancer-related articles, where MeSH (Medical Subject Headings) is the controlled vocabulary of the National Library of Medicine used for indexing PubMed citations (26).

#### Result page

The RLIMS-P result page presents summary statistics of the retrieval results listing the number of documents with potential phosphorylation information (those retrieved based on the applied filter, see Database in Materials and methods) and those with phosphorylation information according to RLIMS-P (i.e. there is at least one substrate identified). The query ‘ “beta catenin” AND cancer [MeSH]’ showed that there were 894 abstracts with potential phosphorylation information, 862 of which were RLIMS-P positive. In addition, the numbers of kinase, substrate and site mentions were displayed, where kinase = 189, substrate = 636 and site = 115 (Figure 1 Statistics). It is important to note that the same query (http://www.ncbi.nlm.nih.gov/pubmed/?term=%22beta+catenin%22+AND+cancer+[MeSH]) in PubMed retrieved 7542 articles, showing that RLIMS-P significantly reduces the number of articles to be inspected.

The RLIMS-P result page (Figure 1 Result table) default view is a summary table where the PMIDs along with the extracted phosphorylated protein and/or kinase names are listed. The Web site offers multiple views of the results summary table, including view by PMID, kinase and substrate (Figure 1d). In all views, documents containing kinase, substrate and site information are listed first. The columns can be sorted using the up and down arrows. The columns are color-coded: green for kinase, blue for substrate and red for site. The results table hides redundant information for a given piece of information to the best extent possible to facilitate data analysis. However, the user can expand the table to view the full results by selecting the ‘plus’ icon next to the option ‘show all annotations’ (Figure 1e). In our example, the intention is to learn about the various kinases phosphorylating beta catenin; therefore, we can use the option view ‘by substrate’ and focus on results where beta catenin is a substrate (Figure 1d). There are 375 unique PMIDs grouped in this category (data not shown). We have drastically reduced the number of articles to inspect (representing around 5% of the 7542 articles in the PubMed search). Review of the information in the results table (Figure 1 Result table) suggests that beta catenin is the substrate for multiple kinases, among them: GSK3B, CK2, PKA, CSNK1A1(CKIalpha), FLT3, PKD1, SRC, PAK1 and PAK4. The results table can be downloaded in CSV format, and the file includes information about the PMID, kinase, substrate and site, along with the extracted sentences containing the relevant evidence.

**Figure 2.**
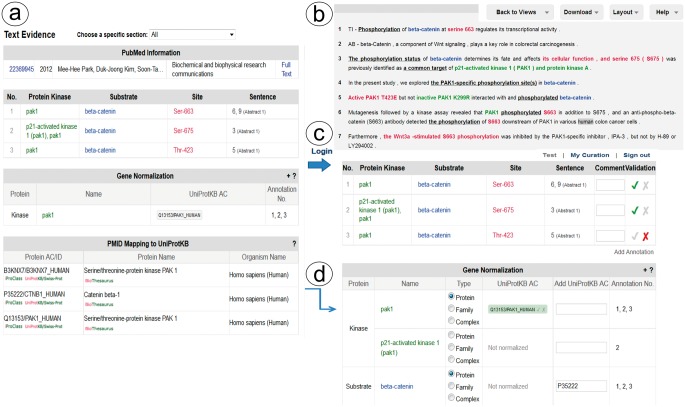
Snapshot of RLIMS-P text evidence page. The text evidence page contains two panels, one containing tables for the article general information, the RLIMS-P annotation, the normalization and UniProtKB mapping (**a**); the other containing the text with color-coded entities (**b**). Once the user logs in, new boxes for editing and validating the annotation and normalization appear (**c**). The information in the mapping table can be used to assist in normalization task during curation (**d**).

Links to the evidence text are provided in three ways: (i) the PMID number in ‘PubMed ID column’ links to NCBI PubMed abstract page, and if a PMCID is also provided in the column, it links to PMC full-text, (ii) the ‘Number of sentences’ column indicates the number of sentences containing evidence for the specific annotation and provides a link to such sentences, (iii) most importantly, selecting the ‘Annotation icon’ (Figure 1f) links to a document-centric page containing the document information, a table with the RLIMS-P results, the text-based evidence with highlighted mentions and the normalization information and mapping to UniProtKB entries, when applicable (Figure 2a). For the example shown, there is information from the UniProtKB mapping about the proteins mentioned in the text. Whenever species names are detected, they are highlighted as requested by users in the IAT, this is the case for the example in Figure 2, where ‘human’ is highlighted in gray (Figure 2b). For each annotation, the section where it was extracted is displayed (Abstract, Results, Figures and/or Discussion), and the user can select what section to review. The information in evidence page can be downloaded in CSV format and BioC format.

#### RLIMS-P validation page

RLIMS-P has editing capabilities to validate, correct, save and download the information. To validate the information in RLIMS-P, the user needs to register and log in (Figure 2c). Once logged in, the validation boxes appear, and the user can check the correct information (see green checks in the example in Figure 2c), and complete information when needed (e.g. the normalization of beta catenin was missing, and in this case, the information in the UniProt mapping helps to fill in the data (Figure 2d)). Importantly, based on user feedback, we added an entity type column in the normalization table to capture annotations of entities such as protein families and complexes, which cannot be normalized to a specific UniProtKB entry.

## Conclusion

The original RLIMS-P system had been used in several curation projects, and has also been used as a component of the eFIP system for mining the functional impact of phosphorylation. The RLIMS-P Web site has been further developed to enhance accessibility of the data extracted by RLIMS-P, and to support practical research and curation procedures, such as search, retrieval, extraction and editing of the phosphorylation information. As demonstrated in the BioCreative IAT, the new Web site makes the large amount of phosphorylation information readily accessible to users. The interface and the functionality of the Web site received highly favorable feedback from curators and other users testing the Web site at BioCreative IV. The extraction performance of the system was good even with the strict evaluation criteria required in the practical curation setting. We have included many of the user suggestions, and the features added not only support practical curation required by biocurators but are also useful for general users. We would like to remark on the potential of RLIMS-P when combined in a working pipeline with other resources. We are collaborating with the PhosphoGrid group, where the curators would use the RLIMS-P system to review and validate the phosphorylation information for yeast, and the annotations would then be exported to the PhosphoGrid pipeline for additional curation/processing steps. RLIMS-P is also used in the iPTMnet resource project (12) (http://proteininformationresource.org/iPTMnet), where literature-mined phosphorylation information is automatically added to the database, and the detected proteins are linked to database identifiers and the phosphorylation sites are validated against the corresponding protein sequence. The integration of the results from RLIMS-P in the iPTMnet adds phosphorylation information from ∼10 000 PMIDs (release 1.0).This illustrates how RLIMS-P can be used in larger curation and resource pipelines.

## Future directions

We are currently working on the improvement of the full-text results presentation in the Web site and the consistency of mentions across the different documents. We will extend RLIMS-P to cover other posttranslational modifications such as ubiquitination, acetylation and glycosylation. We intend to make RLIMS-P results available as a Web service.

## Supplementary data

Supplementary data are available at *Database* Online.
